# Pigeon Pea Intercropped with Tropical Pasture as a Mitigation Strategy for Enteric Methane Emissions of Nellore Steers

**DOI:** 10.3390/ani13081323

**Published:** 2023-04-12

**Authors:** Althieres José Furtado, Adibe Luiz Abdalla Filho, Jaqueline Fernandes Bruno, Rolando Pasquini Neto, Annelise Aila Gomes Lobo, Gabriele Voltareli da Silva, Flavio Perna Junior, Teresa Cristina Alves, Alexandre Berndt, André de Faria Pedroso, Sérgio Raposo de Medeiros, Patrícia Perondi Anchão Oliveira, Paulo Henrique Mazza Rodrigues

**Affiliations:** 1 Faculty of Veterinary Medicine and Animal Science, University of São Paulo, 225 Duque de Caxias North Ave., Pirassununga 13635-900, SP, Brazil; jaqueline.bruno1999@gmail.com (J.F.B.); netopasquini@alumni.usp.br (R.P.N.); anneliselobo@usp.br (A.A.G.L.); gabivoltareli@usp.br (G.V.d.S.); fpernajr@usp.br (F.P.J.); pmazza@usp.br (P.H.M.R.); 2 Embrapa Pecuária Sudeste, km 234 Washington Luiz Highway, ‘Fazenda Canchim’, São Carlos 13560-970, SP, Brazil; teresa.alves@embrapa.br (T.C.A.); alexandre.berndt@embrapa.br (A.B.); andre.pedroso@embrapa.br (A.d.F.P.); sergio.medeiros@embrapa.br (S.R.d.M.); patricia.anchao-oliveira@embrapa.br (P.P.A.O.)

**Keywords:** *Brachiaria* spp., *Cajanus cajan* (L.) Millsp., cattle, greenhouse gas, SF_6_, *Urochloa* spp.

## Abstract

**Simple Summary:**

Intercropping tropical grasses with legumes has the potential to mitigate greenhouse gas emissions in livestock production systems. Here, we evaluate pigeon pea (*Cajanus cajan* (L.) Millsp.) intercropped with *Urochloa* spp. for feeding Nellore steers and compared it with a degraded and recovered pasture system during the rainy and dry seasons of the year. We found that including pigeon pea in grazing systems met the nutritional requirements of the animals to obtain higher gains, improving their performance while also reducing the intensity of enteric CH_4_ emissions, thus contributing to the sustainability of ruminant production based on pastures.

**Abstract:**

In this study, we evaluate the effects of intercropping pigeon pea (*Cajanus cajan* (L.) Millsp.) with tropical pastures for feeding Nellore cattle and compared animal performance and enteric CH_4_ emissions with other pasture-based systems during the dry and rainy seasons of 2021. Thirty-six *Nellore* steers (with a body weight of 221 ± 7 kg and an age of 15–16 months) were randomly distributed in three treatments with three replicates (in paddocks of 1.5 hectares each): (1) a degraded pasture of *Urochloa* spp. (DEG); (2) a recovered and fertilized pasture of *Urochloa* spp. (REC); and (3) pigeon pea intercropped with *Urochloa* spp. (MIX). Enteric CH_4_ emissions were estimated using the sulfur hexafluoride (SF_6_) tracer gas technique, and dry matter intake (DMI) was determined using internal (iNDF) and external (TiO_2_) markers. Forages were collected by hand plucking after observations of ingestive behavior, and feces was collected after voluntary defecation. The proportion of grass and legume intake was estimated by C stable isotopes, and the forage nutritional quality was determined, while animal performance was monitored monthly, and the stocking rate was adjusted by the “put and take” technique. The results indicated that intercropping pigeon pea with tropical grasses is an interesting strategy for sustainable livestock production based on pastures. The MIX treatment was able to meet the nutritional requirements of the animals, which presented higher performance. In addition, there was a reduction in CH_4_ emissions up to 70% when expressed per average daily weight gain in comparison to the DEG treatment.

## 1. Introduction

Agricultural activity is facing many challenges such as the need to increase food production to meet the growing world population [1] while adapting to environmental and economic changes by improving animal performance in more sustainable production systems [2]. Among the issues surrounding the growth of this sector is the increased use and degradation of natural resources, directly contributing to worsening the global climate change scenario due to greenhouse gas (GHG) emissions, depleting water resources, causing soil erosion and impairing natural habitats [3].

Evidence of human-induced climate change and the important contribution of the livestock sector to GHG emissions highlights the need to better understanding the sources of emissions and potential strategies available for their mitigation [3]. Among the GHGs, carbon dioxide (CO_2_), methane (CH_4_), and nitrous oxide (N_2_O) are the most important in the context of the agricultural activity. Although the concentrations of CH_4_ in the atmosphere are lower than those of CO_2_, it has a warming potential that is 27.2 times greater than that of CO_2_ [3]. Additionally, CH_4_ can be classified as a “short lived climate pollutant” having a relatively short lifetime (8–12 years) in the atmosphere compared to CO_2_, which can remain for periods of up to 10,000 years until returning in the global carbon cycle [4].

In this context, special attention should be given to the global livestock production sector, which has been the target of numerous criticisms related to climate change. Brazilian livestock depends on extensive pasture areas, which are often in some stages of degradation, in a production system that emits a high amount of GHGs per unit of produced product [5,6]. Despite this, Brazilian livestock plays a fundamental role in the economy, in which cattle production represents 84% of total animal production (89% beef cattle and 11% milk production) [7]. Brazil has one of the largest herds in the world [8], with approximately 224.6 million heads [9], posing as the world’s largest exporter of beef, with 2.48 million tons in 2021 [10]. In addition, Brazil is the second largest producer of meat, with 9.7 million tons being produced per year, representing 13.7% of world production [10], and in the dairy sector, it is the fourth largest producer of milk, with 35.124 billion liters being produced per year [8].

GHG emissions related to livestock activity in Brazil are of concern. Enteric CH_4_ emissions correspond to 60.1% of the total anthropogenic emissions of this gas, while the fermentation and decomposition of waste correspond to 5.5% of emissions [11]. In 2020, the Brazilian bovine herd was responsible for approximately 5.5% of the total CH_4_ produced worldwide [12].

Aiming to achieve a balance between environment, society, and economy, cattle production previously performed extensively can now be conducted with better planning, making use of management strategies, pasture maintenance, and good agricultural practices that allow livestock activity to be more efficient. This can generate better quality products, with higher production efficiency and reduced damage to the environment [13]. For instance, sustainable intensification results in a less restricted growth trajectory, so negative impacts on meat quality can be avoided and emission intensity can be reduced [14,15].

Among management strategies and agricultural practices, the use of legumes in pastoral systems has great potential to contribute to more sustainable livestock production and to the recovery of degraded pastures. As an example, there is the use of the consortium between pigeon pea (*Cajanus cajan* (L.) Millsp.) and tropical grasses, which could contribute to greater forage production, availability, and quality for feeding the animals, thus reducing the need for nitrogen fertilizers and protein/mineral supplements, especially during the dry season of the year [16,17]. In drier seasons, pigeon pea still has a high capacity to retain leaves after flowering, reaching a production of 12 tons per hectare per year, with its leaves and thinner branches serving as a protein supplement with values of crude protein varying between 16 and 20% [18].

The hypothesis of this study was that the use of pigeon pea in consortium with tropical grasses is an interesting strategy for feeding Nellore cattle, especially in the dry season of the year, contributing to the reduction of GHG emissions, allowing land use intensification and increasing animal productivity. Aiming at achieving a better understanding of the sustainability and environmental consequences of including legumes in tropical pastures, this study evaluates the effects of introducing pigeon pea in tropical grass pastures as an intercropped system to feed Nellore cattle, and thus compares animal production para meters and enteric CH_4_ emissions with other commonly used systems based on pasture, during the dry (May–October) and rainy (November–April) seasons of the year.

## 2. Materials and Methods

### 2.1. Location, Treatments, and Experimental Design

The study was conducted at Embrapa Pecuária Sudeste, São Carlos, SP, Brazil and the treatments consisted of three pasture-based systems: (1) a degraded pasture (DEG) of *Urochloa decumbens* Stapf cv. Basilisk and *U. brizantha* (Hochst ex A. Rich) Stapf cv. Marandu; (2) a recovered pasture (REC) established with a mixture of *U. decumbens* cv. Basilisk and *U. brizantha* (Hochst ex A. Rich) Stapf cv. Marandu; and (3) an intercropped pasture (MIX), which was a mixture of *U. decumbens* cv. Basilisk and *U. brizantha* cv. Marandu intercropped with *Cajanus cajan* (L. Millsp.) cv. BRS Mandarim. Each treatment was arranged in three grazing replicates (in paddocks of 1.5 ha each) in a completely randomized design, totaling to 9 grazing units (13.5 ha in total).

A total of 36 Nellore steers from the herd of Embrapa Pecuária Sudeste (of a body weight of 221 ± 7 kg and an age of 15–16 months) were used as experimental animals randomly distributed in the grazing units (12 animals per treatment—nine noncannulated (tested animals) steers and three rumen-cannulated steers). In each treatment, nine animals were monitored for performance evaluation, six were used for CH_4_ measurements using the SF_6_ tracer gas technique, and three were monitored for dry matter intake (DMI) measurements. The protocols used for the experimental animals followed the guidelines of the Committee for the Use and Care of Institutional Animals (CEUA) of Embrapa (no 05/2016) and College of Veterinary Medicine and Animal Science of University of São Paulo (no 6228200521).

All pastures were managed under continuous grazing, and a variable number of ´nonexperimental´ (regulator) animals were used to adjust the stocking rate by the “put and take” technique as described by Mott and Lucas [19], aiming to maintain a specific intermediate pasture height (of a maximum of 30 cm and a minimum of 15 cm ) as an indirect assessment of forage mass availability [20] in each grazing unit of REC and MIX treatments. When it was not possible to include regulatory animals to adjust the stocking rate, only experimental animals were kept in these pastures. Monthly, the number of animals in each pasture and their weight were monitored to estimate the stocking rate (expressed as the number of animals or animal units (AU) (of a body weight of 450 kg) per ha), animal performance and productivity parameters. The DEG treatment was not managed to maintain a minimum height; however, when the maximum height of the pasture was reached (30 cm), regulator animals were placed in the grazing units. These DEG pastures were classified as degraded using the criterion proposed by Oliveira [21], in which pastures can be considered degraded when there are areas larger than 2 m^2^ in the canopy that are covered by invasive plants, in this case, *Paspalum notatum* Flüggé.

The pastures were established in 1996 with *U. brizantha* cv. Marandu but were later infested by *U. decumbens* cv. Basilisk. In 2011, the area was divided into nine grazing units, and six paddocks randomly converted into REC or MIX treatments. The REC pastures were established by liming, fertilizing and pasture management; moreover, in the MIX pastures, pigeon pea was overseeded and replanted every three years due to the decline in plant population over the years. Currently, the pigeon pea stand is around 180,000 plants per hectare; however, this number may decrease over the years due to animal trampling and grazing, adverse weather conditions, plant senescence, and other reasons [16]. Soil sampling was carried out annually in 0–0.2 and 0.2–0.4 m depths, with 12 subsamples in each paddock (the grazing unit). The REC and MIX pastures received the same application of dolomitic limestone EVN (effective neutralizing value = 70) and fertilization with ordinary superphosphate (OS; 18% of P_2_O_5_) and potassium chloride (KCl; 60% of K_2_O) to reach 70% base saturation, and concentrations of 12 mg dm^−3^ of phosphorus (P) and 3% of potassium (K) in the soil cation exchange capacity [22]. Nitrogen fertilization (200 kg of N-urea per ha, divided into five applications during the rainy season) was applied only for the REC system. The DEG pastures were not corrected nor fertilized.

**Figure 1:**
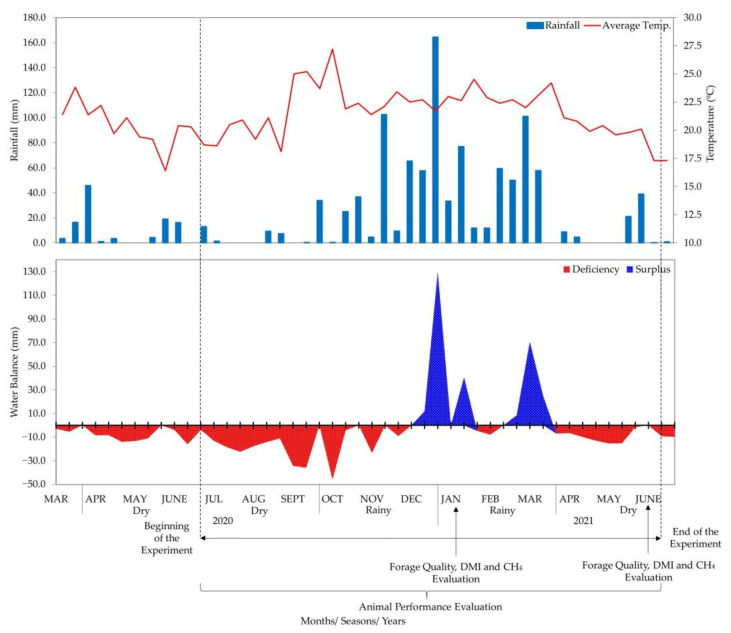
Average temperature (°C), accumulated rainfall (mm) and water balance (mm) throughout the experimental period.

During the experimental period that lasted from July 2020 to July 2021, samples for assessing forage quality, dry matter intake (DMI) and CH_4_ emissions were collected in two seasons: rainy (January) and dry (July). For performance evaluations, the animals were monitored monthly, and the average daily gain (ADG) data was grouped into dry (July–September 2020 and April–June 2021) and rainy (from October 2020 to March 2021) seasons. In the dry season, the average temperature was 20.4 °C and the average cumulative rainfall was 156 mm, while during the rainy season the average temperature was 22.9 °C and the average cumulative rainfall was 868 mm according to the climatic data obtained from an automatic weather station located near the experimental site (http://www.cppse.embrapa.br/meteorologia/index.php?pg=automatica accessed on 23 August 2021) (Figure 1).

A mineral supplement was provided ad libitum throughout the year (Table 1). During the dry season, the MIX animals received the mineral formulation, while the REC and DEG animals received an adaptation formulation for 14 days and then a mineral-energetic-protein supplementation. During the rainy season, all animals received only the mineral formulation.

### 2.2. Forage Sampling and Chemical Analysis

For collecting samples of forages during the two harvest dates of the rainy (January) and dry (July) seasons, the methodology of a grazing simulation with observations of ingestive behavior described by Sollenberger et al. [23] was used. The forage samples (pastures and pigeon pea) were separately collected by hand plucking (±150 g of fresh matter) in three consecutive days, observing the animals for approximately 24 min, and using scissors to cut the portion of forages the animals were consuming. Samples were stored in paper bags (18 cm × 44 cm), weighed, and then dried in a forced ventilation oven at 65 °C for 72 h (pigeon pea samples were dried at 40 °C until the sample weight became constant to not compromise the tannin analysis), milled to 1 mm in a Willey-type mill and subjected to chemical analysis at the Animal Nutrition Laboratory of Embrapa Pecuária Sudeste.

The chemical analysis of the forages was based on the content of DM (DM at 105 °C; Method 934.01, AOAC [24]). Concentrations of mineral matter (MM; Method 923.03), crude protein (CP; Method 920.87) and ether extract (EE; Method 920.85) were determined according to the AOAC [24]. Neutral detergent fiber (NDF), acid detergent fiber (ADF) and lignin (Lig) concentrations were analyzed according to Goering and Van Soest [25]. Gross energy (GE) was determined using a bomb calorimeter (IKA WERKE^®^, model C 500). Condensed tannins (CT) concentrations were evaluated using the methodology proposed by Makkar [26]. The isotope ratio of C (^13^C/^12^C) of the forage samples were determined using a continuous-flow isotope ratio mass spectrometer (Delta Plux, ThermoFisher Scientific^®^, Bremen, Germany) coupled to an elemental analyzer (CHN-1110, Carlo Erba, Rodano, Italy) at the Laboratory of Isotope Ecology of the Center for Nuclear Energy in Agriculture (LEI-CENA/USP), and calculated as:

δ (‰) = [(*R*sample/*R*stantard) − 1] × 1000

where *R* is the ratio of ^13^C/^12^C and Pee Dee Belemnite is the internationally recognized standard.

### 2.3. Dry Matter Intake and Dry Matter Digestibility

The total DMI (kg DM/day) was estimated by the sum of forages and mineral supplements consumed by the animals:

$$DMI={DMI}_{s}+{DMI}_{f}$$

where DMI = total dry matter intake (kg DM/day); DMI_f_ = forage dry matter intake (kg DM/day); DMI_s_ = mineral supplement intake (kg).

The mineral supplement intake was estimated by the difference between the amount provided and the amount of supplement leftovers in the trough after five days. For this measurement, a digital scale (1–10,000 g) was used, and the calculation followed the equation:

$${DMI}_{s}=\frac{\left[\frac{{(DMI}_{sSupplied}-{DMI}_{sLeftovers})}{5_{\left(days\right)}}\right]}{Total\;Weight}$$

where DMI_s_ = mineral supplement intake (kg/kg of BW per day); DMI_sSupplied_ = total supplement provided (kg); DMI_sLeftovers_ = mineral supplement leftovers after 5 days (days); Total Weight = total weight of animals with access to that (kg).

To determine the forage DMI (DMIf), indirect methods with external (titanium dioxide, TiO_2_) and internal (indigestible neutral detergent fiber, iNDF) markers were used. TiO_2_ in small paper capsules was instilled with the aid of an oral applicator. The external marker was administered for 10 days in the amount of 15 g per animal per day. In the last 5 days of TiO_2_ administration, feces samples were collected after spontaneous defecation in the paddocks. The feces samples were frozen in properly identified plastic bags, then thawed, homogenized, and dried at 65 °C for three days.

After drying, the samples were ground in a Willey-type knife mill with 2 mm sieves. Subsequently, an analysis of iNDF and TiO_2_ was performed using the technique described by Mertens [27] and Titgemeyer et al. [28], and DMI_f_ was calculated according to the equation:

$${DMI}_{f}=\frac{\left[{iNDF}_{\left(feces\right)}\times fecal\;output\right]}{{iNDF}_{\left(forages\right)}}$$

where DMI_f_ = forage dry matter intake (kg DM/day); fecal output = TiO_2_ supplied/TiO_2_ recovered in feces (kg/day); iNDF_(feces)_ = feces content of indigestible neutral detergent fiber (%); iNDF_(forages)_ = forage content of indigestible neutral detergent fiber (%).

The dry matter digestibility (DMD) was calculated through an indirect method, using the following equation adapted from Givens et al. [29]:

$$DMD=100-\left(100\times \left(\frac{fecal\;output}{{DMI}_{Total}}\right)\right)$$

where DMD = dry matter digestibility (%); DMI_total_ = total dry matter intake (kg); and fecal output = TiO_2_ supplied/TiO_2_ recovered in feces (kg/day).

Feces samples were also analyzed for their C isotopic composition as previously described, and the principle of isotopic differences between C_3_ and C_4_ plants was used to estimate the intake proportion of each forage (tropical grasses—C_4_; and pigeon pea—C_3_) following the equation described by Norman et al. [30] and Ovani et al. [31]:

$$C_{4}\;(\%)=100-\left(100\times \left(\frac{\left(A-B\right)}{\left(C-B\right)}\right)\right)$$

where A = δ^13^C value in feces; B = δ^13^C value of the C_4_ plant; C = δ^13^C value of the C_3_ plant.

### 2.4. Animal Performance

To determine the performance variables, the animals were weighed on a hydraulic trunk with a built-in scale (Parede Móvel Hidráulico/idBeck 3.0—BechHouser^®^, 2009) after 16 h of fasting, and this was repeated every 28 days. The individual performance was evaluated by the animals’ average daily gain (ADG) obtained by dividing the body weight (BW) difference between two successive weighing measurements performed in an interval of days between the measurements, according to the equation:

$$ADG=\frac{fBW-iBW}{IW}$$

where ADG = average daily gain (kg); fBW = final BW, most recent animal weight (kg); iBW = initial BW, animal weight from previous weighing (kg); IW = interval between weighing (days).

The stocking rate (SR) was expressed in animal units (AUs) and the number of animals per hectare, assuming that one AU is equivalent to 450 kg of animals of the Zebu breed according to the equation:

$$SR=\frac{\left(\frac{BWtotal}{AU}\right)}{Area}$$

where SR = stocking rate (AU ha^−1^); BWtotal = total body weight of tracer and regulator animals present in the experimental area (kg); AU = animal unit (450 kg); Area = experimental unit area (ha).

The feed conversion ratio (FCR), and feed efficiency (FE) were calculated using the following formulations:

$$FCR=\frac{DMI}{ADG}$$

$$FE=\frac{ADG}{DMI}$$

where FCR = feed conversion ratio (kg DMI/kg ADG); DMI = dry matter intake (kg DM/day); ADG = average daily weight gain (kg); FE = feed efficiency (kg ADG/kg DMI).

### 2.5. Enteric CH_4_ Emission

The SF_6_ tracer gas technique [32,33,34,35,36] was used for measuring enteric CH_4_ emissions from rumination, eructation and breathing. Fourteen days before gas sampling, the animals were fitted with gas collection halters to allow acclimatization in an adaptation period. Seventy-two hours prior to the sampling period, a small brass permeation tube was placed in the rumen allowing the tracer gas to equilibrate in the ruminal environment. Each animal was sampled daily (24 h) for five consecutive days. The gas samples were obtained continuously through a capillary tube connected to a collecting container placed on the neck of the animal. A halter with a 0.127 mm stainless steel capillary tube and a 15 μm in-line filter was placed on the animal’s head and connected to an evacuated sampling vessel. Before the experiment, collection canisters made of polyvinyl chloride (PVC) were attached to a vacuum pump in the laboratory to create a negative pressure (of around −13.15 psi). As the vacuum in the sampling vessel slowly dissipated, the negative pressure continuously drew the air sample around the animal’s mouth and nose.

Additional PVC canisters were placed near the experimental pastures to monitor the ambient daily concentration of CH_4_ and SF_6_ during each sampling period. Sampling was performed daily at 07:00 h when the animals were removed from the paddocks and transferred to the working facilities of Embrapa Pecuária Sudeste. After gas sampling, pure nitrogen was added to the canisters and then CH_4_ and SF_6_ were measured using gas chromatographs (Agilent HP-6890, Wilmington, DE, USA; and Shimadzu^®^ GC-2014, Kyoto, Japan).

The CH_4_ flux was calculated following the equation:

$${QCH}_{4}={QSF}_{6}\times \left(\frac{\left[{\left({CH}_{4}\right)}_{Y}-{\left({CH}_{4}\right)}_{b}\right]}{\left[{\left({SF}_{6}\right)}_{Y}-{\left({SF}_{6}\right)}_{b}\right]}\right)$$

where QCH_4_ = CH_4_ emission rate per animal; QSF_6_ = known SF_6_ emission rate from the capsule in the rumen; (CH_4_)_Y_ = CH_4_ concentrations in the collection device; (CH_4_)_b_ = basal concentration of CH_4_; (SF_6_)_Y_ = SF_6_ concentration in the collection device; and (SF_6_)_b_ = basal SF_6_ concentration in the ambient.

The gross energy intake (GEI) was calculated by multiplying the DMI (kg) and diet GE (MJ/kg) content, and the CH_4_ conversion rate (Ym, the percentage of GEI converted to CH_4_) was calculated using the following equation, considering 55.6 MJ/kg the heat value of CH_4_:

$$Ym(\%)=\frac{\left({CH}_{4}\times 55.6\right)}{GEI}\times 100$$

### 2.6. Statistical Analysis

For the statistical analysis, the grazing units (paddocks) were considered the experimental units and data were analyzed using the statistical software SAS 9.4 (SAS Institute Inc., Cary, NC, USA). Before the analysis, outliers were identified, and the normality of residuals was tested (Shapiro–Wilk). When the normality assumption was not accepted, the logarithmic transformation was applied, and then the data were analyzed using the mixed procedure (PROC MIXED) testing different covariance structures and choosing the best fitting model based on the lowest value of the corrected Akaike information criterion (AICC) [37]. The statistical model included the three pasture-based grazing systems and seasons (dry and rainy) as fixed effects, and the interaction between treatment and season was tested. Fixed effects were considered significant at 5%, and in the face of the treatments × seasons interaction the effects of one factor on the other were evaluated using the SLICE command of PROC MIXED. Finally, all means were estimated according to the least squares test (LSMEANS) and the multiple comparisons were performed using the GLIMMIX procedure applying the Fisher’s test through the PDIFF LINES option.

## 3. Results

### 3.1. Pigeon Pea and Tropical Grasses Chemical Composition

The chemical composition and content of condensed tannins of pigeon pea and *Urochloa* spp. sampled during the dry and rainy seasons through hand plucking following the methodology of grazing simulation are presented in Table 2.

The isotopic results (δ^13^C value in feces = −13.8 ± 0.23‰ in the rainy season, and −18.7 ± 1.33‰ in the dry season) indicated that there was little intake of the legume during the rainy season (4.1 ± 0.01); however, during the dry season the intake of pigeon pea reached around 41% (40.7 ± 0.11) of the diet in the MIX treatment. The CP, NDF and ADF content of the forages in the different pasture-based systems, considering the proportion of *Urochloa* spp. (C_4_) and pigeon pea (C_3_) intake for the MIX treatment estimated by stable isotopes are presented in Table 3.

**Figure 2:**
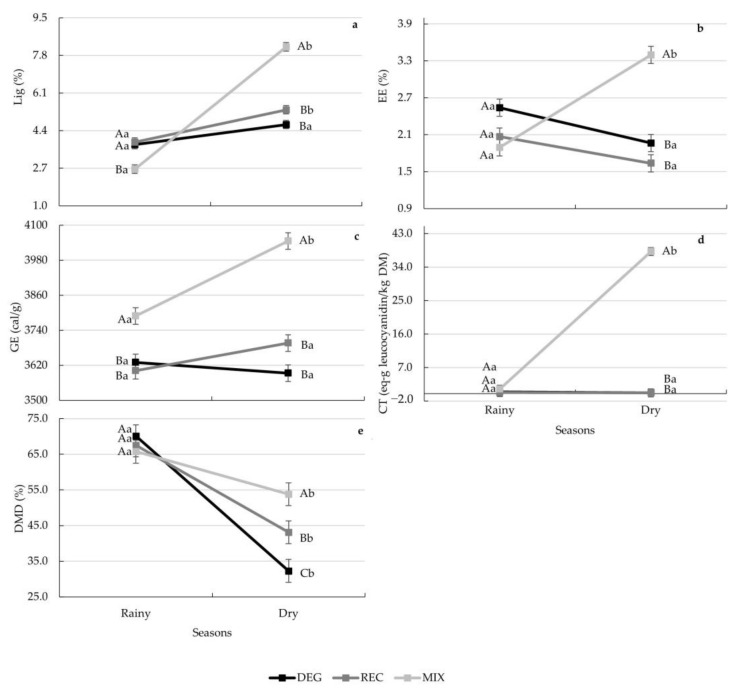
Decomposition of the treatment × season interaction for the lignin (Lig; **a**), ether extract (EE; **b**), gross energy (GE; **c**), and condensed tannin (CT; **d**) contents and dry matter digestibility (DMD; **e**) of the forages collected by hand-plucking in the different pasture-based systems, considering the proportion of *Urochloa* spp. (C_4_) and pigeon pea (C_3_) intake for the MIX treatment estimated by C stable isotopes. Different capital letters indicate statistical differences among treatments in the same season, while different lowercase letters indicate statistical differences between seasons for each treatment by Fisher’s test (*p* < 0.05). Vertical bars represent the standard error of the means.

Treatment affected CP, NDF and ADF, while NDF was also affected by the different seasons (*p* < 0.05). When compared to DEG and REG, the MIX treatment presented 45 and 25% greater values of CP (*p* = 0.0016). Additionally, MIX presented approximately 12% lower values of NDF (*p* = 0.0013) and 14% lower values of ADF (*p* = 0.0012) compared to the other treatments. During the dry season, a 10% lower value of NDF was found compared to the rainy season (*p* = 0.0004). The decomposition of the treatment × season interactions is present in Figure 2. Similar results of EE, CT and DMD were found among the treatments during the rainy season (Figure 2b,d,e) (*p* > 0.05). However, during the dry season, greater values of these variables were found for MIX compared to the REC and DEG treatments (Figure 2b,d,e) (*p* < 0.05). During both seasons, MIX presented greater values of GE compared to the REC and DEG treatments (Figure 2c) (*p* = 0.0343). The Lig content in the MIX treatment was lower in the rainy season and greater in the dry season compared to REG and DEG (*p* < 0.0001) (Figure 2a).

### 3.2. Forage and Mineral Supplement Intake

The average values of the forage and mineral supplement DMI during the experimental period are presented in Table 4. When the DMI was expressed as %ABW, no effect of treatment (*p* > 0.05) was found for the forage, total, and supplement DMI. Nevertheless, when expressed as kg DM/day, a 43% lower supplement DMI was found in the MIX treatment (*p* = 0.0068). In addition, when expressed as kg DM/day, 70% higher values of forage (*p* = 0.0118) and total DMI (*p* = 0.0132) were found in the rainy season, while when expressed as %ABW, a supplement DMI of 2.4 times greater was found in the dry season (*p* = 0.0420).

### 3.3. Animal Performance and Stocking Rate

The average values of iBW, fBW, ADG, FCR and FE in the three different pasture-based treatments in the rainy or dry seasons during the experimental period are presented in Table 5. Approximately 16 and 9% greater values of fBW (*p* = 0.0165), and 57 and 21% greater values of ADG (*p* = 0.0008) were found in the MIX treatment compared to DEG and REC. The rainy season presented a 72% lower value of FCR (*p* = 0.0175). A FE value of three time greater (*p* = 0.0063) and an ADG value of six times greater (*p* <.0001) were found during the rainy season. In fact, all treatments presented greater ADG values in the rainy season, and the MIX treatment showed greater ADG value (*p* = 0.0546) compared to DEG and REC in both seasons.

**Figure 3:**
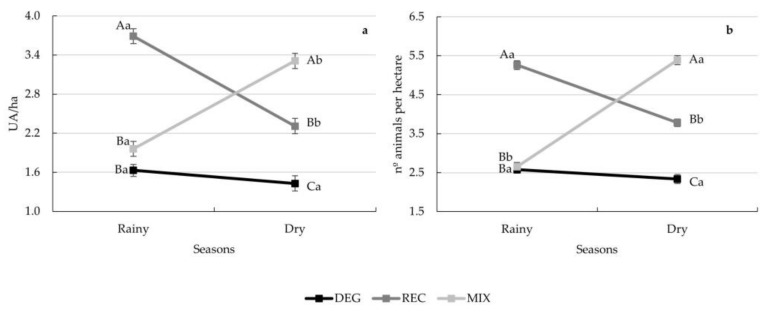
Decomposition of the treatment × season interaction for stocking rate expressed as UA per hectare (**a**) and as number of animals per hectare (**b**) in the different pasture-based systems during the experimental period. Different capital letters indicate statistical differences among treatments in the same season, while different lowercase letters indicate statistical differences between seasons for each treatment by Fisher’s test (*p* < 0.05). Vertical bars represent the standard error of the means.

Interactions between season and treatments were found for the stocking rate parameters (*p* < 0.0001) (Figure 3). For the MIX treatment, a higher stocking rate value was found in the dry season of the year (*p* < 0.0001), which could be related to the greater forage biomass usually obtained when including *Cajanus cajan* (L.) Millsp. in pasture systems. However, during the rainy season, the highest stocking rate was found in the REC treatment (*p* < 0.0001).

### 3.4. Enteric CH_4_ Emissions

The average values of enteric CH_4_ emissions per hectare, AU and GEI using the SF_6_ tracer gas technique in the different treatments and seasons, as well as the statistical pro-abilities, are presented in Table 6. No effect of treatment or season was found when expressed per hectare and AU (*p* > 0.05). When expressed as CH_4_/GEI, 59% greater emission was found in the rainy season of the experimental period (*p* = 0.0212).

**Figure 4:**
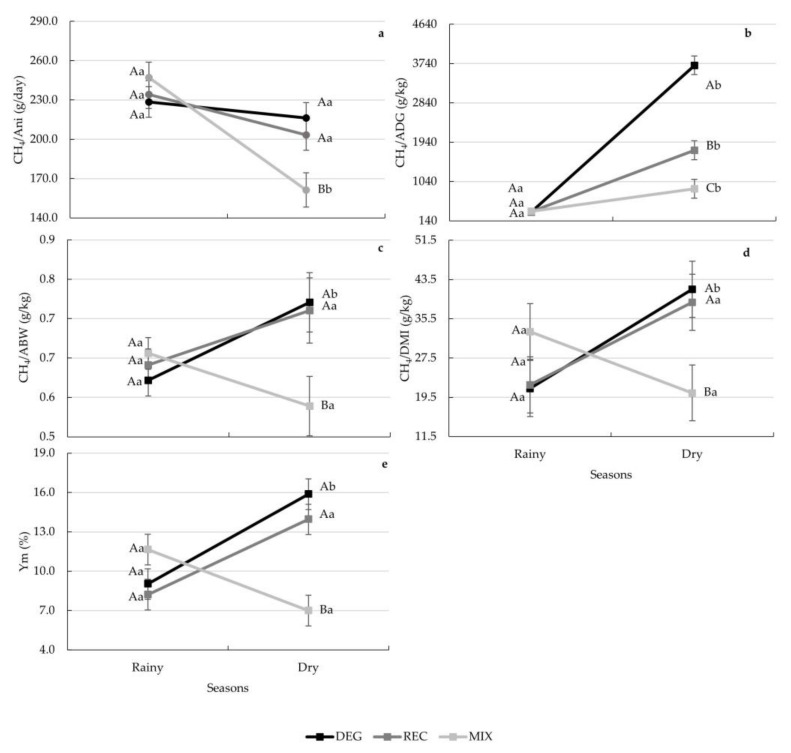
Decomposition of the treatment × season interaction for enteric CH_4_ emissions (**a**–**d**) and Ym (**e**) in the different pasture-based systems during the experimental period. Different capital letters indicate statistical differences among treatments in the same season, while different lowercase letters indicate statistical differences between seasons for each treatment by Fisher’s test (*p* < 0.05). Vertical bars represent the standard error of the means.

Interactions between season and treatments were found for the enteric CH_4_ emission parameters and Ym (*p* < 0.05) (Figure 4a–e). No differences among treatments were found in the rainy season of the experimental period (*p* > 0.05). During the dry season, lower values of enteric CH_4_ emissions and Ym were found for MIX compared to both the DEG and REC treatments (*p* < 0.05) (Figure 4a–e). Additionally, during the dry season, lower emissions of CH_4_ per ADG were found for REC compared to the DEG treatment (*p* > 0.05) (Figure 4b). In comparison to the DEG treatment, there was a reduction in CH_4_ emissions in the MIX treatment of up to 70% when expressed per ADG during the dry season of the experimental period (3699.62 vs. 873.04 g CH_4_/kg ADG; Figure 4b).

## 4. Discussion

The hand plucking technique was used for separately sampling the forage in the different treatments, and the isotopic analysis allowed the estimation of the proportion of *Urochloa* spp. and pigeon pea intake in the MIX treatment. A recent review article by Castro-Montoya and Dickhoefer [17] pointed out that there are 18 in vivo trials with pigeon pea being fed to ruminants, and to the best of our knowledge, this is the first study reporting the nutritional quality of a diet composed by *Urochloa* spp. and pigeon pea in an intercropped pasture-legume system for feeding *Nellore* cattle in Southeast Brazil.

Efficient digestion by ruminal microorganisms requires at least 7% of CP [38], and during both seasons the CP values of all treatments were above the minimum and were consistent with the values reported for fertilized and unfertilized *Urochloa* spp. pastures [39]. Additionally, the CP content of *Cajanus cajan* (L.) Millsp. sampled during both dry and rainy seasons (Table 2) were in line with those reported by Miano et al. [40], Hampel et al. [41] and Valadares Filho et al. [42] (17 to 24% CP), resulting in an average CP content of the MIX treatment being 25–45% greater than that of the REC and DEG during the experimental period, which may have contributed to the better animal performance of this treatment. In forage diets, the NDF content is one of the determinants of forage intake [43], and the DEG and REC treatments showed NDF and ADF values in line with those reported for *Urochloa* spp. under tropical conditions [39,44,45]. For the MIX treatment, NDF content was lower than those found for pastures intercropped with pigeon pea [41]. Additionally, the lowest NDF and ADF values of the MIX treatment were in line with those reported by Alves et al. [46] and Pereira et al. [47] when evaluating the nutritional quality of *Cajanus cajan* (L.) Millsp. However, the value of ADF in the MIX treatment was higher than that reported for a consortium of *Panicum maximum* Jacq. and pigeon pea [41]. On the other hand, the Lig values of the MIX treatment were lower than those found for this consortium [41]. The mean EE values of the DEG and REC treatments were similar to those reported by Sá et al. [48] for C_4_ pastures composed mainly of *Urochloa* spp, and the EE values of MIX treatment are similar to those found by Vitti et al. [49] and Castro-Montoya and Dickhoefer [17] when evaluating the nutritional quality of pigeon pea. In both seasons, the GE content of DEG and REC treatments were similar to those found by de la Mora et al. [50], while the GE of the MIX treatment was similar to that for a pigeon pea green forage in the Brazilian Tables of Feed Composition for Cattle [42].

In both seasons, the CT content of the DEG and REC treatments were higher than the content of other tropical grasses reported by Bueno et al. [51], while the MIX treatment presented values higher than those found by Pereira et al. [47] for pigeon pea, and values lower than those found in a tropical pasture intercropped with pigeon pea [41]. Some studies have shown that feed consumption by ruminants can be reduced when the concentration of TC exceeds 50 g CT/kg DM, due to the reduction in acceptability and conditioned aversion [52,53]. As the level of CT found here for all treatments was below this value, no negative effect was seen on the consumption of the diet, as other authors have shown when using diets with similar CT contents, irrespective of the plant used [54,55,56,57]. In addition, according to Perna Junior et al. [58], values of around 20 to 45 g CT/kg DM are sufficient to interfere in the digestive process of ruminants.

The DMD of the MIX treatment during the dry season was higher than that reported for a pigeon pea green forage [42], while the DMD value of REC was similar to those found by Dias et al. [59] and Euclides et al. [60]. During the dry season the DMD of MIX was greater than that of both the DEG and REC treatments, a similar result to that found by Epifanio et al. [61] when evaluating *Urochloa* spp. intercropped with the legume *Stylosanthes* spp., which revealed an increase in digestibility compared to pastures composed only with grasses. A possible explanation for the higher DMD value of MIX is some of the associative effects between forages on feed digestion [62]. Increased digestion when a low-quality forage is supplemented by a legume with high nitrogen content can be attributed to the stimulation of the microbial activity and modification of digestive processes in the rumen, including proteolysis and CH_4_ production when secondary metabolites such as tannins, saponins or polyphenol oxidase are present in low quantities [62].

During the dry season, the forage and total DMI were lower than those found in the rainy season. These results can be justified by the structure of the vegetation, lower acceptability, presence of antinutritional compounds, lower passage rate of food through the gastrointestinal tract and lower forage availability in the dry season of the year, in addition to factors inherent to the animals such as breed, sex and age [63,64]. The DMI of REC was lower than that found by Meo-Filho et al. [65]. For all treatments, the DMI during the rainy season was approximately 1 kg lower than that described by Barioni et al. [66] in DMI tables for *Nellore* steers under grazing conditions. In the dry season, the DMI values for all treatments were similar to those reported by Barioni et al. [66]. In a meta-analytical approach evaluating zebu animals grazing *Urochloa* spp. with mineral and energy/protein supplementation [67], the DMI results were lower than those found in this study, with the average performance of animals consuming only mineral supplementation being similar to those found in the DEG treatment. In addition, energy/protein supplement consumption was around 1 kg per animal [67], a value above that found in the DEG and REC treatments. The weight gain of the animals receiving the energy/protein supplement, in amounts of around 580 g per day, was greater than that found in the treatments of this study.

Daily DMI is a very important factor in ensuring the release of nutrients for maintenance and production. Tulu et al. [68] found considerable variations in DMI among pigeon pea genotypes. Usually during the dry season, tropical grasses present low nutritional quality and forage availability, and these could explain the lower forage and total DMI found in this study during this season. Additionally, in the dry season, animals preferentially consume more supplements to enhance the use of diet substrates and optimize animal performance and feed efficiency by ameliorating the pasture’s nutritional composition [69,70], and a higher intake of the supplement was found during the dry season when expressed as %ABW. However, despite the different composition of mineral supplements throughout the year among the treatments, a lower supplement DMI was found for the pasture with pigeon pea (MIX). This could be attributed to some of the pigeon pea characteristics since it is a legume that reaches its reproductive phase and improved acceptability of its pods and oldest leaves during the dry season of the year, being consumed as an important source of protein [16,71], thus reducing the need for energetic-protein mineral supplements [72]. In times of scarcity and high prices for protein mineral supplements, the introduction of this legume in pasture systems is even more relevant.

The similar iBW evidenced the animals’ weight uniformity among the treatments, while higher fBW and ADG values in the MIX treatment compared to DEG and REC indicate greater performance in the pasture intercropped with pigeon pea. A higher performance of cattle on pastures intercropped with legumes was also found by Machado and Sales [73] when comparing these to pastures with *Urochloa* spp exclusively. Both forage DMI and ADG values were in line with those described by Oliveira et al. [16] for a consortium system using pigeon pea.

It is important to consider that pigeon pea can fix N content and add organic matter to the soil, factors that can contribute to greater forage nutritional quality and availability to the animals. This legume also contributes to the recovery of degraded pastures [16], which represent approximately 70% of pasture areas in Brazil [74,75]. In the DEG treatment, which represents a pasture with some level of degradation, the stocking rate expressed both as the number of animals per hectare and AUs per hectare was lower than that in the other treatments, a fact that could be related to the low persistence and biomass production of the tropical grass in a soil without proper nutritional management [39]. The REC treatment that received nitrogen fertilization showed a higher stocking rate during the rainy season, with values similar to those found by Meo-Filho et al. [65] when evaluating a fertilized intensively managed pasture under rotational grazing with a liming application. However, during the dry season, the REC treatment had a lower stocking rate than MIX. During dry seasons, the seasonality of production and nutritional quality of tropical grasses are observed [63,64], reducing a pasture’s support capacity, while it is in this period that pigeon pea begins to be consumed more as an important source of forage for animals, enabling a higher stocking rate [16]. Considering the seasons, a higher feed conversion ratio was found during the dry season, and this is justified by the poorer nutritional quality of the forages. In the same way, the greater feed efficiency found in the rainy season is justified by the better nutritional quality of the forage to which the animals had access during this season [76].

Decreasing the emissions of enteric CH_4_ from ruminant production is a strategy to limit the global temperature increase to 1.5 °C by 2050 [77]. During the dry season, when higher pigeon pea intake was observed, CH_4_ emissions expressed per animal, per ADG, per ABW and per DMI were lower in the MIX treatment, which can be attributed to some of its nutritional quality and CT content. The effect of tannins on the reduction in enteric CH_4_ production is usually related to its direct action by inhibiting the activity of methanogenic microorganisms and/or reducing the digestibility of rumen fiber fractions [78]. Additionally, it is important that the benefits of the reduction in the emission of CH_4_ do not hide the possible harmful effects of tannins on nutrient digestibility and production parameters [79]. Further in vitro studies using tannin-binding agents (e.g., polyethylene glycol) evaluating the effects of pigeon pea on diet degradability, ruminal fermentation parameters, ruminal microorganisms and the potential of CH_4_ mitigation may contribute to elucidating the results found in this study. In this sense, Berhanu et al. [80] evaluated the in vitro potential of mitigating CH_4_ emissions from several legumes, including pigeon pea, and found a lower production of total gases as well as of CH_4_.

When expressed per ADG, the highest CH_4_ emission was found for DEG during the dry season of the year, which can be explained by the reduced performance results of this treatment. During the dry season, the MIX treatment showed higher performance results, which contributed to the lower emission intensity found in the system with the inclusion of pigeon pea. When expressed as a percentage of the gross energy intake (Ym), similar values among treatments were found during the rainy season. However, in the dry season, the lowest Ym was found in the treatment with pigeon pea, once again indicating the potential that this intercropped system has in contributing to the sustainability of livestock production based on pastures.

Finally, the results of this study highlight the fact that the inclusion of pigeon pea in pasture-based systems can represent an advantage not only for cattle farmers in raising animals with greater performance, but also for Brazil as a country, which made a commitment to reduce CH_4_ emissions by 30% by 2030 during the 26th UN Climate Change Conference of the Parties (COP26), in Glasgow, Scotland.

## 5. Conclusions

The introduction of pigeon pea in tropical pastures as an intercropped system used to feed *Nellore* cattle was able to meet the nutritional requirements of the animals in the MIX treatment. In this treatment, the animals presented a lower intake of the mineral supplement, greater average daily gain, and reduced intensity emissions of enteric CH_4_ compared to other pasture-based systems commonly used in Brazil. These results confirmed our initial hypothesis that pigeon pea intercropped with tropical grasses may improve the sustainability of livestock production based on pastures, especially in the dry season of the year, contributing to the reduction of GHG emissions and increasing animal productivity. Future research may consider the inclusion of this legume during backgrounding and finishing beef cattle on tropical pastures, as well as its effects on the ruminal environment, potential carcass production and meat quality.

## Figures and Tables

**Table 1:** Formulation and composition of the mineral supplements.

Supplements				
Ingredients	Mineral ^4^	Mineral-Energetic-Protein	Mineral-Energetic-Protein	
		Adaptation ^3^	Dry Season	
Ground corn (%)	-	55	48	
NaCl (%)	50	20	15	
Mineral mixture ^1^ (%)	50	15	15	
Urea ^2^ (%)	-	10	22	
Estimated Chemical Composition				
CP (%)	-	49.33	97.15	
NPN (%)	-	28	61.6	
NDF (%)	-	5.01	7.30	
ADF (%)	-	1.53	1.67	
Lig (%)	-	1.26	1.25	
EE (%)	-	1.66	1.29	
Ash (%)	90.68	56.6	22.60	

^1^ Minerthal^®^ quantity per kg of product: 200 g of calcium, 160 g of phosphorus, 60 g of sulfur, 185 g of sodium, 200 mg of cobalt, 2.5 g of copper, 1.6 g of fluorine, 125 mg of iodine, 2.25 g of manganese, 50 mg of selenium, and 7.5 g of zinc; ^2^ Heringer^®^; ^3^ adaptation supplement provided for 14 days for adaptation of rumen microbiota; ^4^ mineral formulation conducted for MIX throughout the year and in the rainy season for REC and DEG; CP: crude protein; NPN: nonprotein nitrogen; NDF: neutral detergent fiber; ADF: acid detergent fiber; Lig: lignin; EE: ether extract; Ash: mineral matter.

**Table 2:** Chemical composition and condensed tannin content of *Cajanus cajan* (L.) Millsp. and *Urochloa* spp. separately sampled in the MIX treatment during the rainy and dry seasons of the experimental period.

Plant Species	Seasons	CP	NDF	ADF	Lig	EE	GE	CT (eq g Leucocyanidin/kg DM)	δ^13^C (‰)
		(%)	(%)	(%)	(%)	(%)	(cal/g)		
*Cajanus cajan* (L.) Millsp.	Rainy	17.8	42.4	26.8	12.3	5.7	4431.3	23.7	−30.5
	Dry	24.3	43.9	28	12.4	5.6	4509.8	87.9	−26.1
	Average	21	43.2	27.4	12.4	5.7	4470.5	55.8	−28.3
	SEM	1.77	0.82	0.43	0.38	0.14	94.27	15.11	0.68
*Urochloa* spp.	Rainy	10.2	65.7	36	2.2	1.7	3744.8	0.2	−13.2
	Dry	8.6	67.4	40.6	5.1	1.8	3749	1.6	−13.7
	Average	9.4	66.6	38.3	3.7	1.7	3746.9	0.9	−13.4
	SEM	0.50	0.58	1.14	0.66	0.15	41.06	0.43	0.10

Crude protein (CP), neutral detergent fiber (NDF), acid detergent fiber (ADF), lignin (Lig), ether extract (EE), mineral matter (Ash), neutral detergent insoluble nitrogen (NDIN), acid-detergent insoluble nitrogen (ADIN), gross energy (GE) expressed as calories per gram (cal/g) and condensed tannins (CT), expressed as eq g leucocyanidin/kg DM (dry matter), and carbon isotope ratio (δ^13^C). SEM: standard error of the mean.

**Table 3:** Crude protein, neutral detergent fiber and acid detergent fiber contents of the forages in the different pasture-based systems, considering the proportion of *Urochloa* spp. (C_4_) and pigeon pea (C_3_) intake for the MIX treatment estimated by C stable isotopes.

Effects		Nutritive Composition		
Treat.	Seasons	CP	NDF	ADF
		(%)	(%)	(%)
DEG		7.9 ^b^	71.4 ^a^	40.1 ^a^
REC		9.2 ^b^	69.7 ^a^	40.5 ^a^
MIX		11.5 ^a^	61.0 ^b^	35.4 ^b^
	Rainy	9.6	70.9	38.6
	Dry	9.5	63.4	38.8
Average		9.5	67.4	38.7
SEM		0.40	0.80	0.70
Statistical Probabilities (*p*-value)				
Treat.		0.0016	0.0013	0.0012
Seasons		0.7907	0.0004	0.8563
Treat. × Season		0.2206	0.8416	0.8131

^a,b^ Different lowercase letters in the same column represent treatments that differ from each other (*p* < 0.05) by Fisher’s test. Crude protein (CP), neutral detergent fiber (NDF), acid detergent fiber (ADF). DEG: degraded pasture of *Urochloa* spp.; REC: *Urochloa* spp. pasture fertilized with 200 kg of N-urea ha^−1^ year; MIX: *Urochloa* spp. pasture intercropped with *Cajanus cajan* (L. Millsp.) cv. BRS Mandarim. SEM: standard error of the mean.

**Table 4:** Average values of forage, mineral supplement and total DMI in the different pasture-based systems during the experimental period.

Effects		Variables						
Treat.	Seasons	Forage DMI		Supplement DMI		Total DMI		Total DMI/BW^0.75^
		(kg/day)	(%ABW)	(kg/day)	(%ABW)	(kg/day)	(%ABW)	(kg/kg)
DEG		7.12	2.33	0.07 ^a^	0.023	7.20	2.42	0.103
REC		7.56	2.20	0.07 ^a^	0.025	7.63	2.27	0.102
MIX		8.24	2.29	0.04 ^b^	0.015	8.28	2.32	0.102
	Rainy	9.62	2.59	0.05	0.011	9.67	2.64	0.120
	Dry	5.66	1.96	0.07	0.027	5.73	2.04	0.084
Average		7.64	2.27	0.06	0.021	7.70	2.29	0.102
SEM		1.650	0.240	0.005	0.0020	0.830	0.230	0.0100
Statistical Probabilities (*p*-value)								
Treat.		0.7689	0.9503	0.0068	0.2158	0.7856	0.9492	0.9922
Seasons		0.0118	0.0903	0.0552	0.0420	0.0132	0.0965	0.0544
Treat. × Season		0.3145	0.2009	0.1384	0.9399	0.3245	0.2088	0.5642

^a,b^ Different lowercase letters in the same column represent treatments that differ from each other (*p* < 0.05) by Fisher’s test. DMI: dry matter intake; ABW: average body weight; BW^0.75^: metabolic body weight. DEG: degraded pasture of *Urochloa* spp.; REC: *Urochloa* spp. pasture fertilized with 200 kg of N-urea ha^−1^ year; and MIX: *Urochloa* spp. pasture intercropped with *Cajanus cajan* (L. Millsp.) cv. BRS Mandarim. SEM: standard error of the mean.

**Table 5:** Average values of initial and final body weight, average daily gain, feed conversion ratio and feed efficiency in the different pasture-based systems and seasons of the experimental period.

Effects		Variables				
Treat.	Seasons	iBW	fBW	ADG	FCR	FE
		(kg)	(kg)	(kg)	(kg/kg)	(kg/kg)
DEG		220.7	344.1 ^b^	0.304 ^c^	37.9	0.038
REC		221.3	367.4 ^b^	0.393 ^b^	50.5	0.043
MIX		220.2	401.9 ^a^	0.478 ^a^	28.5	0.062
	Rainy	*	*	0.671	17.2	0.073
	Dry	*	*	0.113	60.2	0.022
Average		220.7	371.1	0.392	38.8	0.048
SEM		8.10	9.80	0.01	8.10	0.009
Statistical Probabilities (*p*-value)						
Treat.		0.9952	0.0165	0.0008	0.4768	0.4135
Seasons		*	*	<0.0001	0.0175	0.0063
Treat. × Season		*	*	0.0546	0.4708	0.5642

^a,b,c^ Different lowercase letters in the same column represent treatments that differ from each other (*p* < 0.05) by Fisher’s test. * Data not presented by season. iBW: initial body weight; fBW: final body weight; ADG: average daily gain; FCR: feed–conversion ratio; FE: feed efficiency. DEG: degraded pasture of *Urochloa* spp.; REC: *Urochloa* spp. pasture fertilized with 200 kg of N-urea ha^−1^ year; MIX: *Urochloa* spp. pasture intercropped with *Cajanus cajan* (L. Millsp.) cv. BRS Mandarim. SEM: Standard error of the mean.

**Table 6:** Average values of CH_4_ emissions and Ym in the different pasture-based systems and seasons of the experimental period.

Effects		Variables		
Treat.	Seasons	CH_4_/ha	CH_4_/AU	CH_4_/GEI
		(kg/ha)	(kg/AU)	(MJ)
DEG		84.1	73.23	108.7
REC		141.0	66.30	115.4
MIX		121.1	63.87	137.0
	Rainy	122.5	66.83	147.7
	Dry	108.3	68.77	93.0
Average		115.38	67.80	120.4
SEM		10.810	3.340	14.60
Statistical Probabilities (*p*-value)				
Treat.		0.0606	0.3623	0.5222
Seasons		0.4169	0.7192	0.0212
Treat. × Season		0.4854	0.1248	0.2683

CH_4_/ha: methane emissions by hectare; CH_4_/AU: methane emissions by animal unit (450 kg of body weight), CH_4_/GEI: methane emissions by gross energy intake. DEG: degraded pasture of *Urochloa* spp.; REC: *Urochloa* spp. pasture fertilized with 200 kg of N-urea ha^−1^ year; MIX: *Urochloa* spp. pasture intercropped with *Cajanus cajan* (L. Millsp.) cv. BRS Mandarim. SEM: standard error of the mean.

## Data Availability

The data presented in this study are available upon request from the corresponding author.
